# STRsearch: a new pipeline for targeted profiling of short tandem repeats in massively parallel sequencing data

**DOI:** 10.1186/s41065-020-00120-6

**Published:** 2020-03-16

**Authors:** Dong Wang, Ruiyang Tao, Zhiqiang Li, Dun Pan, Zhuo Wang, Chengtao Li, Yongyong Shi

**Affiliations:** 1 Bio-X Institutes, Key Laboratory for the Genetics of Developmental and Neuropsychiatric Disorders (Ministry of Education), Collaborative Innovation Center for Brain Science, Shanghai Jiao Tong University, Shanghai, China; 2 Shanghai Key Laboratory of Forensic Medicine, Shanghai Forensic Service Platform, Academy of Forensic Science, Ministry of Justice, Shanghai, 200063 People’s Republic of China

**Keywords:** Short tandem repeats, Massively parallel sequencing, STR genotyping, Validation studies, Forensic sequencing

## Abstract

**Background:**

Short tandem repeats (STRs) are important polymorphism makers for human identification and kinship analyses in forensic science. With the continuous development of massively parallel sequencing (MPS), more laboratories have utilized this technology for forensic applications. Existing STR genotyping tools, mostly developed for whole-genome sequencing data, are not effective for MPS data. More importantly, their backward compatibility with the conventional capillary electrophoresis (CE) technology has not been evaluated and guaranteed.

**Results:**

In this study, we developed a new end-to-end pipeline called STRsearch for STR-MPS data analysis. The STRsearch can not only determine the allele by counting repeat patterns and INDELs that are actually in the STR region, but it also translates MPS results into standard STR nomenclature (numbers and letters). We evaluated the performance of STRsearch in two forensic sequencing datasets, and the concordance with CE genotypes was 75.73 and 75.75%, increasing 12.32 and 9.05% than the existing tool named STRScan, respectively. Additionally, we trained a base classifier using sequence properties and used it to predict the probability of correct genotyping at a given locus, resulting in the highest accuracy of 96.13%.

**Conclusions:**

All these results demonstrated that STRsearch was a better tool to protect the backward compatibility with CE for the targeted STR profiling in MPS data. STRsearch is available as open-source software at https://github.com/AnJingwd/STRsearch.

## Background

Short tandem repeats (STRs) are short tandemly repeated DNA sequences composed of repetitive units of 1–6 bp [1]. STRs are widespread throughout the human genome and serve as widely used polymorphism markers in forensic science [1, 2]. For forensic casework, ideal STR loci should generally have the following characteristics such as approximate fragments ranging from 100 to 500 bp, high heterozygosity, low stutter, a low mutation rate, and so on [3, 4]. Currently, the capillary electrophoresis (CE) technology is the gold standard for STR genotyping, and it is commonly used in national DNA databases. The main process of the CE method includes PCR amplification of multiple STR loci, STR allele separation and sizing, and profile interpretation [3, 5, 6]. Each STR amplicon has been fluorescently labeled during PCR, and then STR alleles are separated via gel or CE based on dye color and migration time. Finally, compared to the allelic ladder with calibrated repeat numbers, the number of repeats of each allele is determined [3]. However, the CE method can only identify length variation and does not account for any sequence variation in repeat or flanking regions [7].

Compared to the CE method, massively parallel sequencing (MPS) can not only analyze an increased number of STR loci simultaneously, but it also provides higher discrimination power by detecting various sequence variants such as SNPs or INDELs [8]. However, there are three main difficulties in developing new tool for STR-MPS data analysis: (i) the amplification of STR loci during sequencing is also subject to slippage, creating copy number errors in read data; (ii) the low information content of repetitive sequence reads makes it difficult to align them reliably [9]; (iii) existing bioinformatics tools, mostly can make reliable calls only if sequencing reads completely span the actual repeat region [10].

For the first challenge, these errors are usually termed as stutters, which are commonly encountered artifacts during STR analysis both in CE and MPS data. They are caused by the slippage of the DNA polymerase during the extension phase of the PCR, generating the deletion or extra one repeat unit in the nascent DNA strand [11]. For the second challenge, a previous study [10] performed a comprehensive survey and then demonstrated that Stampy [12] was the most accurate with regards to mapping reads in STR regions, while Novoalign (http://www.novocraft.com), Bowtie2 [13] and BWA [14] consumed much shorter running times. For the third challenge, research demonstrated that long-read sequencing technologies (such as Nanopore or PacBio) could potentially sequence through larger repeat loci with accuracy and effective cost [15]. Furthermore, short paired-end reads with sequence overlaps can be assembled to create longer sequences, and assembled reads will span the full length of the original DNA fragment.

So far, for STR analysis in whole-genome sequencing data, many tools have been developed, the most notable of which are LobSTR [16], HipSTR [17] and RepeatSeq [18]. However, the capacity of these tools was severely restricted to detecting STR variation within read length. To solve this problem, another tool called STRetch [19] estimated the approximate size of STR allele using the normalized read counts that were linearly related to the length. For targeted profiling of STRs, STRScan [20] identified STRs by comparing read sequences with repeat patterns. However, the priori assumption on allele size had the potential to induce allelic dropout. While STRaitRazor [21] adopted approximate string matching of flanking sequences to characterize haplotypes of STRs. So sufficient and unique flanking sequences were required to allow them to be mapped correctly. Although the importance of internal and external quality control (QC) was highlighted for STRs analysis by many guidance papers [22–24], the QC process was not included in currently accessible tools.

In addition to the difficulties mentioned above, there are three crucial challenges to implement MPS in forensic genetics, including a lack of consistent nomenclature and reporting standards, a lack of compatibility with existing National DNA Database infrastructure, and a lack of population data to support statistical calculations [25]. In the past years, to accelerate the progress towards a consistent and platform-independent nomenclature system, the STR sequence template file [26] and the forensic STR sequence guide [27] were dynamically revised and released. These detailed annotations for STR are vital to produce correct genotypes in MPS data and protect their backward compatibility with vast STR data produced by the CE method.

In this study, based on the most up-to-date STR annotations, a new end-to-end pipeline named STRsearch is proposed for targeted profiling of STRs in MPS data. The STRsearch pipeline is implemented and packaged using Python, supporting both versions of Python: 2 and 3. It is freely available and can be downloaded from https://github.com/AnJingwd/STRsearch. Meanwhile, this application is also released using Docker, and the Docker image is published in Docker Hub (https://hub.docker.com/r/anjing123/strsearch), so it is easier to pull the image (with the command “docker pull anjing123/strsearch”) and run it by a container on your local machine.

## Methods

### The massively parallel sequencing datasets

We tested STRsearch in two MPS datasets: one produced by Ion *S5*™ System, with BAM-files (the original sequence data) containing single-end reads [28], whereas the other obtained via Illumina *MiSeq* platform, with 2 × 250 bps paired-end reads (not published). In each of these datasets, the panel is composed of STR loci which are commonly used in DNA forensics: the first dataset (denoted as the Ion *S5* dataset) including 31 STRs from autosomes (excluded 4 gender determination loci), and the other dataset (denoted as the Illumina *MiSeq* dataset) consisting of 58 autosomal STRs, 6 X-chromosome STRs, and 23 Y-chromosome STRs. At the same time, genotyping results on each STR locus using the CE method were obtained for the two different panels, only excluding two genotypes with no call in the Illumina *MiSeq* dataset. The detailed descriptions of the CE genotyping method and analytical threshold were provided by previous studies [28, 29].

### The STRsearch pipeline

**Fig. 1:**
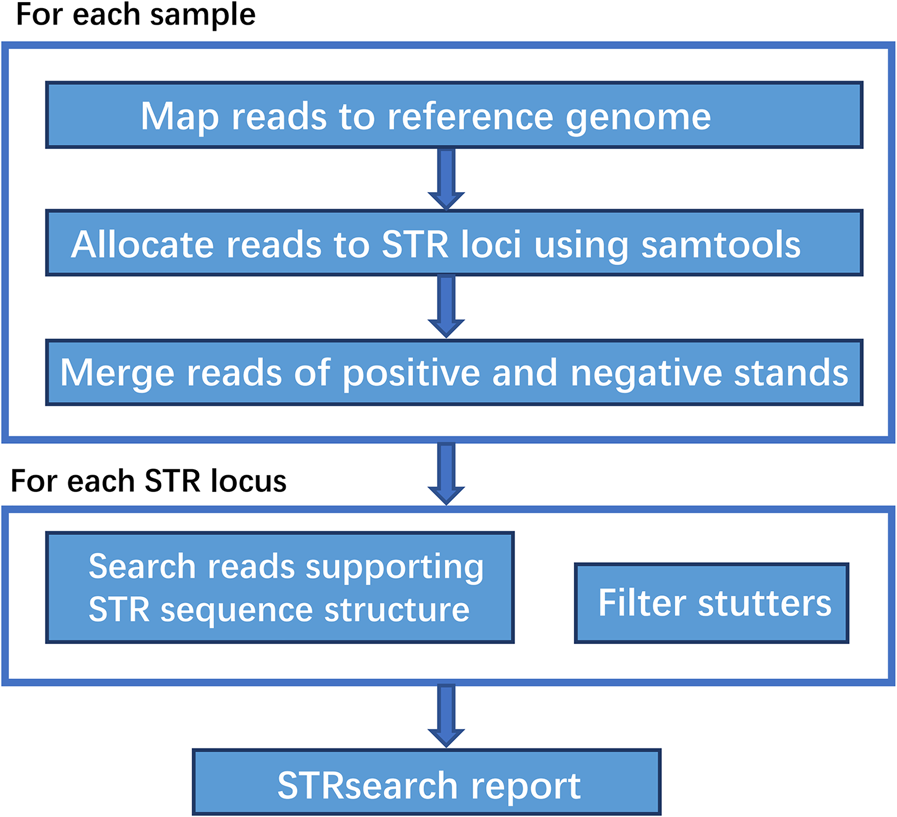
Summary of the STRsearch pipeline. The resulting report consists of three tables of genotyping results, multiple alleles, and a quality control matrix on each STR locus

In brief, to determine the STR allele supported by a read, the STRsearch pipeline employs the strategy of counting repeat patterns and INDELs that are actually in the repeat region. In the meantime, the MPS results are translated into the standard STR nomenclature (numbers and letters). Generally, the pipeline consists of three major components with functions, including allocating reads to STR loci, searching motifs of STR nomenclature, and stutters filter according to allele frequencies. Meanwhile, a parallel architecture is adopted by Python multiprocessing module for very high-speed performance. A summary of the STRsearch pipeline is presented in Fig. 1, with further specifics detailed below.

### Editing a configuration file for STR loci

For targeted profiling of STRs in MPS data with STRsearch, the first step is to create a configuration file about a user-defined panel of STR loci. It is indispensable to provide STR’s repeat region in a reference genome, STR nomenclature, the original reading sequence of STR (forward or reverse), 5′ and 3′ flanking sequences. Note that flanking sequences are necessarily adjacent to STR repeat region. It is recommended that STR nomenclature should be described according to the latest revised forensic STR sequence guide [27] from the STRidER database [30]. In brief, STR repeat structure is described as following rules [27]: (1) repeating elements (usually termed motifs) are bracketed followed by the suffix ‘n’ to signify a repeat number; (2) non-repetitive but counted motifs are not bracketed and are given in upper case; (3) nucleotide tracts that don’t need to be counted are given in continuous lower case; (4) motifs or uncounted tracts are separated by a single space. The detailed guidance and example for editing the standard configuration file can be found from the STRsearch repository at GitHub.

### Aligning and allocating reads to STR loci

The STRsearch is designed to handle both single-end and paired-end data. If FASTQ data provided, reads are mapped to the reference genome sequence using BWA-MEM [14] (v1.7) and SAMtools [31] (v1.7) (Python script: bwa_align.py). Otherwise, STRsearch provides an option of directly handling the BAM-file and skipping the first mapping step. According to the location of STR repeat region, STRsearch then allocates likely STR reads from the BAM-file for each STR locus using SAMtools view, respectively. For paired-end data, on the basis of the original reading sequence, a DNA sequence on the opposite strand is converted into its reverse-complement counterpart using seqtk (https://github.com/lh3/seqtk) (v1.2-r94), and then reads are combined for next STR analysis (Python script: get_STR_fastq.py). Additionally, to obtain longer reads for STR analysis, STRsearch provides an option to assemble paired-end reads using fastq_mergepairs command of USEARCH [32] package.

### STR calling based on STR nomenclature

First of all, STRsearch extracts DNA strings of all motifs and information of which motifs should be counted from STR nomenclature. At the next moment, STRsearch adopts a three-step strategy to address the STR profiling problem (Python script: STR_search.py). Firstly, all motifs are respectively aligned to a read with no mismatch permitted using a dynamic programming algorithm. Only if there is no exact match to any position of the read for all motifs, this read will be discarded. Secondly, an iterative algorithm is applied to obtaining the longest continuous interval composed of all motifs. Finally, the STR sequence extends on both sides to the place where flanking sequences can be aligned with minimal mismatches. To get comparable results to CE calls, all DNA bases (insertions or deletions) that are actually in the STR region will be counted to determine the allele supported by a read. For example, if the insertion fragment surrounding by leading and trailing flanking sequences is [TTCC]15TT, the repeat number will be called as 15 by comparing the STR motif (TTCC) with reads alone, and as 15.2 by the STRsearch, since there is a length difference.

### Stutter filter and allele report

Based on allele frequencies, the statistical evaluation of STR genotypes is performed, and multiple alleles are classified into STRs, stutters, and noise (Python script: STR_parse.py). For STR loci from the autosomal chromosomes, two alleles present with the two most supported reads. The stutter ratio is defined as a ratio of allele frequencies between the second allele to the most frequent one. Generally, the peak height ratio (also termed as heterozygote balance) of 0.5~0.6 is routinely accepted for the CE method [33]. Just et al. [34] verified the performance of the ForenSeq™ system (the first MPS-STR assay) and lowered the STR intra locus balance threshold from the default 60 to 50% to address quality issues. Therefore, the default analytical threshold of stutter ratio is 0.5 in STRsearch, and when the ratio is lower than 0.5, the second allele is regarded as a stutter. For STR locus that is carried by X or Y chromosome of the male sample, we know that only a single allele presents and that reads supporting an allele besides the modal allele will be identified as stutters. Noise is defined as the low-frequency alleles that make up no more than 1% of total alleles on each locus. It may be PCR or sequencing errors and is removed from the multi-allele report of STRsearch. In order to filter genotypes with bias, a quality control matrix is output, including several sequence properties (total bases, sequencing quality score, number of allocated reads, distance distribution of STR sequence to end of reads, and allele read depth).

## Results

### STRsearch is able to recover true STR genotypes

Before STR genotyping, the configuration file required by STRsearch pipeline was edited based on the latest forensic STR annotation, which was downloaded from the website, https://strider.online/bundles/strbaseclient/downloads/Forensic_STR_Sequence_Structure_Guide_v5.xlsx [27]. After sequencing data was mapped to the *Homo sapiens* reference genome with BWA, STRsearch extracted reads overlapping each identified targeted STR locus, respectively. Subsequently, the repeat number of STR alleles was reported and STR sequences were translated into the simple STR nomenclature (numbers and letters) (see Methods). To make results comparable and protect the backward compatibility with the CE method, it was fundamental for different tools to calculate the allele size based on the consistent and latest STR annotation of the forensic STR sequence guide. Because it was convenient to convert the latest STR nomenclature to patterns defined by STRScan with minimal modifications. The other popular STR profiler named STRScan was chosen and we ran it with default parameters to estimate the size of the short allele in the Ion *S5* datasets. At the same time, CE genotyping results were used as ground truth for comparing STRsearch and STRScan genotyping results.

In the Ion *S5* dataset, a further concordance study was performed between STRsearch calls and CE genotypes for all 50 individual samples (including a positive control sample 9947A) and all 31 STRs, resulting in the evaluation of 1550 loci (Additional file 1: Table S1). On average, STRsearch reported 30.4 STR genotypes per sample (range 29–31). The additional 34 genotypes listed as “NA” (no call) were excluded from the comparison, and a further investigation suggested that no reads were extracted for these genotypes on two STR loci (Penta D and Penta E). In total, 1516 comparable genotypes identified by STRsearch included 1148 (75.73%) genotypes in concordance with CE, 368 (24.27%) genotypes in discordance at a 0.5 stutter ratio. In general, there were three types of discordances: (i) 35 (9.51%) genotypes were incorrectly parsed as homozygous genotypes owing to a higher stutter ratio than 0.5. (ii) 183 (49.73%) genotypes showed much smaller allele size than CE results. By checking quality control matrix output by STRsearch and BAM-files with Integrative Genomics Viewer (IGV), we found that the 3′-end of reads on four loci (FGA, D18S51, D19S433, and Penta D) didn’t span STR repeat regions, resulting in truncated allele sequences. (iii) some markers showed consistent differences of one or more repeat units between STRsearch and CE, likely due to annotation differences, and needed to be corrected before performing comparisons. Table 1 contains comparison results between STRsearch and CE for 9947A in the Ion *S5* dataset.

**Table 1:** Comparison results between STRsearch and CE for 9947A in the Ion *S5* dataset

Marker	STR sequence sturucture^1^	Alleles (a1, a2)	Supporting reads (a1, a2)	Alleles correction^2^ (a1, a2)	Allele sequences (a1, a2)	CE
D1S1677	[TTCC]n	13, 14	592, 498	–	[TTCC]13, [TTCC]14	13, 14
D1S1656	CCTA [TCTA]n	18.3, 19.1	1182, 378	18.3, 18.3	CCTA [TCTA]13 TCATCTATCTATCTATCTA, CCTA [TCTA]13 TCATCTATCTATCTATCTACA	18.3, 18.3
TPOX	[AATG]n	8, 7	2554, 95	8, 8	[AATG]8, [AATG]7	8, 8
D2S441	[TCTA]n	10, 14	1007, 874	–	[TCTA]8 TCTGTCTA, [TCTA]11 TTTATCTATCTA	10, 14
D2S1776	[AGAT]n	10, 9	2825, 172	10, 10	[AGAT]10, [AGAT]9	10, 10
D2S1338	[GGAA]n GGAC [GGAA]n [GGCA]n	19, 23	718, 715	–	[GGAA]12 [GGCA]7, [GGAA]2 GGAC [GGAA]13 [GGCA]7	19, 23
D3S1358	[TCTA]n [TCTG]n [TCTA]n	15, 14	2052, 1916	–	[TCTA]1 [TCTG]2 [TCTA]12, [TCTA]1 [TCTG]2 [TCTA]11	14, 15
D3S4529	[GATA]n AATA [GATA]n	12, 11	1886, 65	12, 12	[GATA]4 AATA [GATA]7, [GATA]4 AATA [GATA]6	13, 13
D4S2408	[ATCT]n	10, 9	98, 56	–	[ATCT]10, [ATCT]9	9, 10
FGA	[GGAA]n GGAG [AAAG]n AGAA AAAA [GAAA]n	7.5, 8.5	693, 27	7.5, 7.5	[GGAA]2 GGAG [AAAG]4 AGAA, [GGAA]2 GGAG [AAAG]5 AGAA	23, 24
D5S2800	[GGTA]n [GACA]n [GATA]n [GATT]n	14, 23	1130, 876	–	[GGTA]3 [GACA]6 [GATA]2 [GATT]3, [GGTA]9 [GACA]6 [GATA]3 [GATT]5	14, 23
D5S818	[ATCT]n	11, 11	2373, 295	11, 11	[ATCT]11, [ATCT]11 T	11, 11
CSF1PO	[ATCT]n	10, 12	1348, 1178	–	[ATCT]10, [ATCT]12	10, 12
D6S1043	[ATCT]n	12, 18	1693, 1263	–	[ATCT]12, ATCTATCTATCTATCTATCTATGT [ATCT]12	12, 18
D6S474	[AGAT]n [GATA]n	14, 18	1898, 1304	–	[AGAT]5 [GATA]9, [AGAT]5 [GATA]13	13, 17
D7S820	[TATC]n	10, 11	1133, 823	–	[TATC]10, [TATC]11	10, 11
D8S1179	[TCTA]n [TCTG] n [TCTA]n	13, 13	1382, 998	–	[TCTA]1 [TCTG]1 [TCTA]11, [TCTA]13	13, 13
D10S1248	[GGAA]n	13, 15	815, 811	–	[GGAA]13, [GGAA]15	13, 15
TH01	[AATG]n ATG [AATG]n	8, 9.3	1728, 1527	–	[AATG]8, [AATG]6 ATG [AATG]3	8, 9.3
vWA	[TAGA]n [CAGA] n TAGA	17, 18	1330, 952	–	[TAGA]12 [CAGA]4 TAGA, [TAGA]13 [CAGA]4 TAGA	17, 18
D12S391	[AGAT]n GAT [AGAT] n [AGAC]n AGAT	18, 20	1171, 846	–	[AGAT]11 [AGAC]6 AGAT, [AGAT]12 [AGAC]7 AGAT	18, 20
D12ATA63	[TTG]n [TTA]n	13, 12	1697, 214	13, 13	[TTG]3 [TTA]10, [TTG]3 [TTA]9	13, 13
D13S317	[TATC]n	11, 10	2216, 177	11, 11	[TATC]11, [TATC]10	11, 11
D14S1434	[CTGT]n [CTAT]n	11, 13	1418, 1094	–	[CTGT]3 [CTAT]8, [CTGT]3 [CTAT]10	11, 13
Penta E	[TCTTT]n	12, 13	443, 425	–	[TCTTT]12, [TCTTT]13	12, 13
D16S539	[GATA]n	11, 12	2293, 1661	–	[GATA]11, [GATA]12	11, 12
D18S51	[AGAA]n	5, 3	2030, 159	5, 5	[AGAA]5, [AGAA]3	15, 19
D19S433	[CCTT]n ccta [CCTT] n cttt [CCTT]n	8, 7	859, 485	–	[CCTT]8, [CCTT]7	14, 15
D21S11	[TCTA]n [TCTG]n [TCTA]n ta [TCTA]n tca [TCTA]n tccata [TCTA]n TA [TCTA]n	30, 29	1450, 166	30, 30	[TCTA]6 [TCTG]5 [TCTA]3 ta [TCTA]3 tca [TCTA]2 tccata [TCTA]11, [TCTA]6 [TCTG]5 [TCTA]3 ta [TCTA]3 tca [TCTA]2 tccata [TCTA]10	30, 30
Penta D	[AAAGA]n	4, 3	206, 22	4, 4	[AAAGA]4, [AAAGA]3	12, 12
D22S1045	[ATT]n ACT [ATT]n	11, 14	1033, 616	–	[ATT]8 ACT [ATT]2, [ATT]11 ACT [ATT]2	11, 14

^1^Reference sequence repeat region sequence structure summary based on the most up-to-date forensic STR sequence guide

^2^Alleles correction according to the stutter ratio, which is 0.5 in this study. ‘-’, not applicable

When STRScan was performed in the same dataset, the sample No.614 was removed because STRScan program aborted with a core dumped error.

For the remaining 49 samples, STRScan just reported an average of 27.4 genotypes (range 26–28) and failed in searching for any reads supporting the STRs on three loci (vWA, D12S391, D19S433) in all samples (Additional file 2: Table S2). The concordance study of STRScan with CE showed 851 (63.41%) genotypes in concordance at a 0.5 stutter ratio among a total of 1342 valid genotypes. However, we found that STRScan could not correctly count allele size of non-standard motifs, for example, the widely observed TH01 9.3 allele was therefore improperly genotyping as 9 by STRScan. Compared to STRsearch, more discordances on account of stutter ratio were observed (57 genotypes) that might suggest that STRScan was more sensitive on identifying sequencing reads supporting STRs. Table 2 includes comparison results between STRScan and CE for 9947A in the Ion *S5* dataset.

**Table 2:** Comparison results between STRScan and CE for 9947A in the Ion *S5* dataset

Marker	Repeat motif^1^	Alleles (a1, a2)	Supporting reads (a1, a2)	Alleles correction^2^ (a1, a2)	CE
D1S1677	(TTCC)15	13, 14	1323, 1103	–	13, 14
D1S1656	(CCTA)1(TCTA)16	19, 18	2493, 532	19, 19	18.3, 18.3
TPOX	(AATG)8	8, 7	4552, 184	8, 8	8, 8
D2S441	(TCTA)12	10, 14	2134, 1825	–	10, 14
D2S1776	(AGAT)11	10, 9	5559, 320	10, 10	10, 10
D2S1338	(GGAA)2(GGAC)1(GGAA)13(GGCA)7	23, 22	752, 95	23, 23	19, 23
D3S1358	(TCTA)1(TCTG)1(TCTA)14	15, 14	3054, 2966	–	14, 15
D3S4529	(GATA)4AATA(GATA)7	11, 10	2713, 120	11, 11	13, 13
D4S2408	(ATCT)9	10, 9	993, 776	–	9, 10
FGA	(GGAA)2GGAG(AAAG)14AGAAAAAA(GAAA)3	20, 21	229, 124	–	23, 24
D5S2800	(GGTA)3(GACA)8(GATA)3(GATT)3	14, 13	1615, 66	14, 14	14, 23
D5S818	(ATCT)11	11, 10	3734, 426	11, 11	11, 11
CSF1PO	(ATCT)14	10, 12	2442, 1996	–	10, 12
D6S1043	(ATCT)12	12, 18	3563, 2567	–	12, 18
D6S474	(AGAT)5(GATA)12	14, 18	3063, 2052	–	13, 17
D7S820	(TATC)13	10, 11	1544, 554	10, 10	10, 11
D8S1179	(TCTA)1(TCTG)1(TCTA)11	13, 12	2665, 275	13, 13	13, 13
D10S1248	(GGAA)13	15, 13	1327, 1057	–	13, 15
THO1	(AATG)7ATG(AATG)0	9, 9	3298, 122	9, 9	8, 9.3
vWA	(TAGA)11(CAGA)5TAGA	NA	0, 0	–	17, 18
D12S391	(AGAT)11(AGAC)7AGAT	NA	0, 0	–	18, 20
D12ATA63	(TTG)3(TTA)10	12, 11	3478, 417	12, 12	13, 13
D13S317	(TATC)11	11, 10	3516, 196	11, 11	11, 11
D14S1434	(CTGT)3(CTAT)10	11, 13	2236, 1679	–	11, 13
Penta E	(TCTTT)5	13, 12	629, 624	–	12, 13
D16S539	(GATA)11	11, 12	4105, 3085	–	11, 12
D18S51	(AGAA)18	15, 19	1300, 934	–	15, 19
D19S433	(CCTT)12cctaCCTTctttCCTT	NA	0, 0	–	14, 15
D21S11	(TCTA)4(TCTG)6(TCTA)3ta(TCTA)3tca(TCTA)2tccata(TCTA)11	30, 29	2833, 301	30, 30	30, 30
Penta D	(AAAGA)13	12, 13	53, 1	12, 12	12, 12
D22S1045	(ATT)14ACT(ATT)2	10, 13	2109, 1390	–	11, 14

^1^Reference sequence repeat region sequence structure based on the latest forensic STR sequence guide with modifications to meet requirements of STRScan

^2^Alleles correction according to the stutter ratio, which is 0.5 in this study. ‘-’, not applicable

Furthermore, the utility of STRsearch for STR analysis was validated in the Illumina *MiSeq* dataset with relatively longer reads. We compared STRsearch calls for the dataset to the CE genotypes. The remaining 3666 genotypes in 50 samples were evaluated after filtering out untyped genotypes, resulting in 75.75% concordance. In order to get unique genotypes from STRScan with paired-end sequencing data, we combined alleles found on the positive and negative strands. By contrast, the overall concordance was 66.70% among 3601 comparable genotypes for STRScan call sets in the same dataset.

### Building a base classifier with quality control matrix

**Fig. 2:**
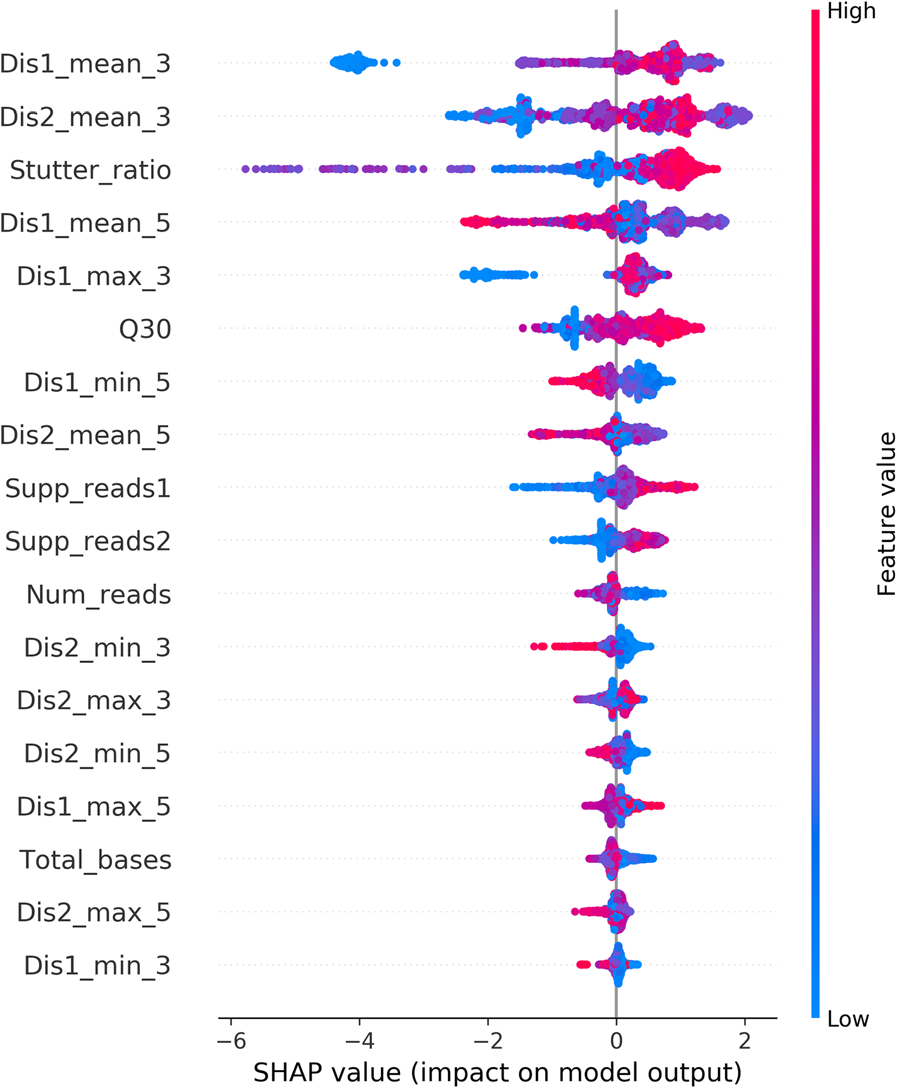
A distribution plot of impacts each feature has on the model output. The color represents the feature value (red high, blue low). This reveals, for example, that a short mean distance between the first allele sequence end and read 3′-end (Dis1_mean_3) lowers the predicted probability of correct genotyping

In order to filter STRsearch calls to obtain only high-quality genotypes, a base classifier using the XGBoost algorithm [35] was built. In the Ion *S5* dataset, the quality control matrix reported by STRsearch (see methods) was used as features, and the concordance with CE was used as labels (Additional file 3: Table S3). We used k-fold cross-validation and selected the best model with the best parameters, resulting in a predicting accuracy of 96.13% in the validation set. The results indicated that the model could predict the probability that reflected the accuracy of calls. Furthermore, we used SHAP (SHapley Additive exPlanations) [36] to interpret predictions of the model, the results showed that mean distance of STR sequence to the 3′-end of reads was the most important feature, following by stutter ratio (Additional file 4: Fig. S1). The impact of different feature values on the output of the model is displayed in Fig. 2. It was consistent with the knowledge that low-quality bases of 3′-end were usually trimmed, resulting in shorter reads than STR repeat region. Then we also tested the performance of the model in the Illumina *MiSeq* dataset, while the accuracy was 75.65%. On the one hand, it might be due to some causes of discordances (such as STR annotation differences) that cannot be characterized by the QC matrix. On the other hand, it suggested that this model might differ between sequencing platforms (i.e., Illumina vs. Ion Torrent) or also between different protocols (PCR free vs. not).

## Discussion

Over the past several decades, because of the rapid development of MPS, the forensic community has ushered in the opportunity to conduct STR variation analysis with this technology. In spite of the advent of MPS, it is indispensable for forensic casework to make sequence-based STR genotype compatible with CE data populating all national DNA databases. A consistent and platform-independent nomenclature system has been addressed by the International Society of Forensic Genetics (ISFG) [26]. In the foreseeable future, these recommendations will be generally embraced and used in practical applications of forensic genetics.

Here, based on the latest STR annotation of the system, an end-to-end pipeline called STRsearch is developed for STR-MPS data analysis. Briefly, STRsearch employs an iterative algorithm to obtain the longest continuous interval, which is composed of all motifs of STR nomenclature. In the process of comparing the read sequence and single motif, it is not needed for a priori assumptions on the repeat number, which is STRsearch’s advantage over the existing tool named STRScan. The actual STR region is determined by comparing the position of repeat patterns with the best matching location of flanking sequences in reads. Therefore, there is an improvement in detecting reliable STR genotypes over matching the leading and trailing flanking regions alone. Because variants or PCR errors in flanking sequences will make it difficult to locate the repeat region of STR locus. Ultimately, allele size is calculated not only for repeat patterns but also for INDELs that are actually in the STR region. This counting way was demonstrated to get more consistent results with the CE method. For paired-end sequencing, STRsearch provides an option to assemble paired-end reads to create relatively longer sequence information for STR analysis. Because of the 100~500 bp STR loci commonly used in forensics, this strategy is sufficient when sequencing data is produced by the Illumina *MiSeq* platform. Moreover, STRsearch has several key features of a simple configuration process, high-speed performance, and convenient deployment.

The main limitation of STRsearch is that it is built upon mapping tools to allocate reads to STR loci, and thus may not be sensitive enough. However, this limitation is mostly relevant to the effectiveness of alignment method for STR regions. In addition, STRsearch does not include modules for calling SNPs or INDELs, because alignments produced by BWA-MEM may not be entirely reliable.

## Conclusion

In this paper, we present STRsearch, which allows the targeted profiling of STRs in MPS data. It perfectly supports the most up-to-date and CE-compatible nomenclature of the forensic STR sequence guide. Therefore, it is beneficial for STRsearch to obtain a compatible CE allele call plus a simple STR nomenclature (numbers and letters) for MPS results. Comparing to existing tools named STRScan, STRsearch showed improved concordance with CE genotypes at specific loci, as a priori assumptions on allele size is not required. Therefore, STRsearch is a better tool for targeted STR profiling in MPS data.

## Availability and requirements

Project name: STRsearch.

Project home page: https://github.com/AnJingwd/STRsearch

Operating system(s): Linux.

Programming language: Python.

Other requirements: bwa v1.7 or higher, samtools v1.7 or higher, bamToFastq v2.17.0 or higher, seqtk v1.2 or higher, usearch v11 or higher.

License: MIT.

Any restrictions to use by non-academics: no restrictions.

## Supplementary information

**Additional file 1 Table S1.** Comparison results between STRsearch and CE in 50 samples.

**Additional file 2 Table S2.** Comparison results between STRScan and CE in 49 samples.

**Additional file 3 Table S3.** The quality control matrix reported by STRsearch and CE in 50 samples.

**Additional file 4 Figure S1.** A bar graph of feature importance ranking for sequence properties used in a base classifier.

## Data Availability

The datasets used and analyzed during the current study are available from the corresponding author on reasonable request.

## Abbreviations

CE: Capillary electrophoresis
INDELs: Insertions and deletions
MPS: Massively parallel sequencing
PCR: Polymerase chain reaction;
QC: Quality control
SNPs: Single nucleotide polymorphisms
STRs: Short tandem repeats
