# Supplementary figures and images for: STRsearch: a new pipeline for targeted profiling of short tandem repeats in massively parallel sequencing data
Source: Hereditas. 2020 Mar 16;157:8. doi: 10.1186/s41065-020-00120-6 (PMC7075041; doi:10.1186/s41065-020-00120-6)

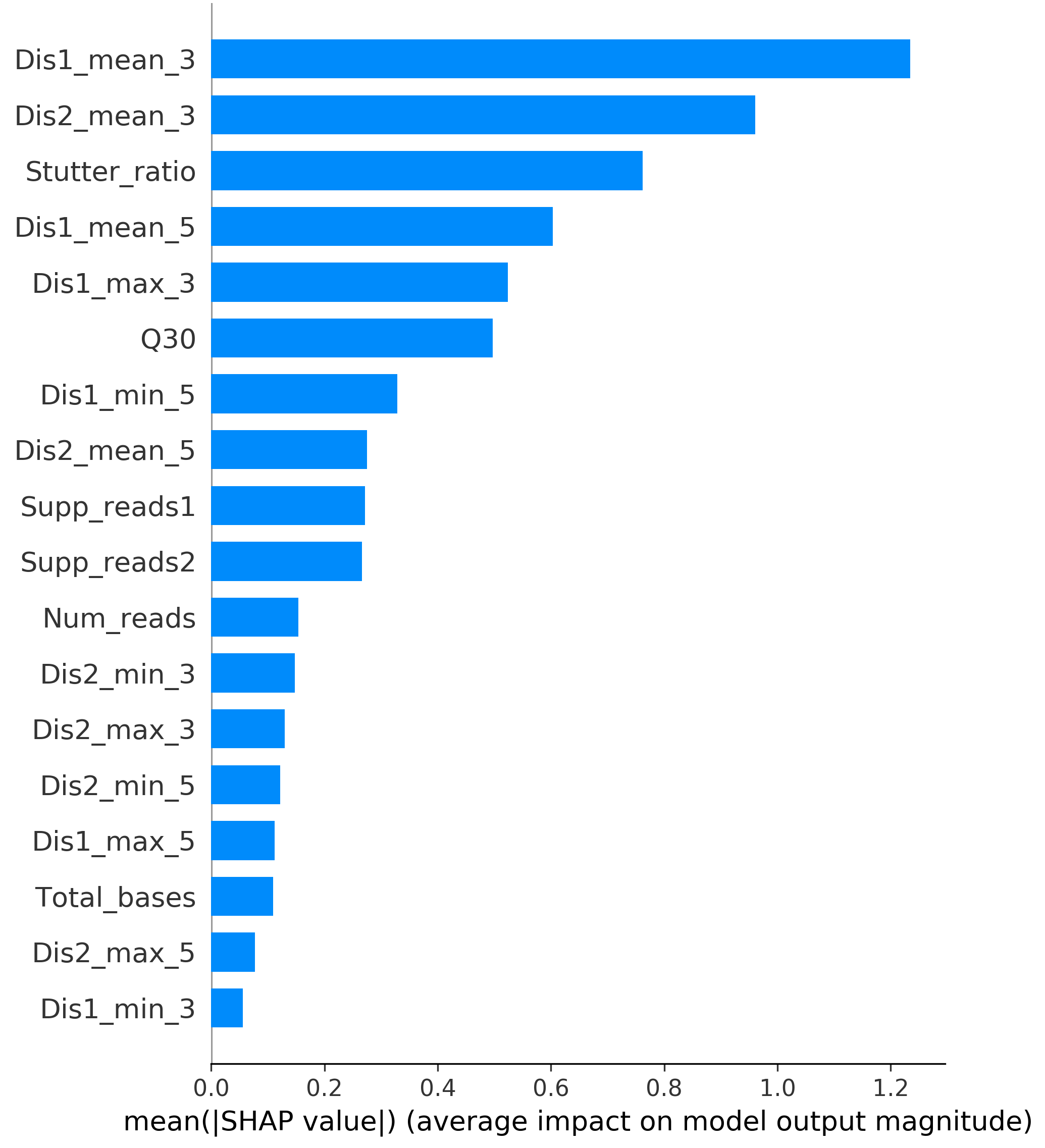

Supplement: Supplementary file 4 — Additional file 4 Figure S1. A bar graph of feature importance ranking for sequence properties used in a base classifier. [file 41065_2020_120_MOESM4_ESM.tif]
